# Supplementary material for: Examining attitudes about the virtual workplace: Associations between zoom fatigue, impression management, and virtual meeting adoption intent
Source: PLoS One. 2025 Feb 5;20(2):e0312354. doi: 10.1371/journal.pone.0312354 (PMC11798524; doi:10.1371/journal.pone.0312354)
Supplement: S1 File — Questionnaire Items Used For this Study. (DOCX) [file pone.0312354.s001.docx]

**Appendix 1. Questionnaire Items Used For this Study**

All response options of all measures are 5 Likert-type scales ranging from 1, “Not at all” to 5 “Extremely,” unless we present a different option. Participants were asked to answer the most commonly used VM platform they use at the beginning of the survey among the possible response options ranging from Zoom, Google Meets, Gather.town, Facetime, etc. Once they answer this question, their response (e.g., Zoom) was tailored to ask the following questions. The questions for each measure included the phrase ‘the VM platform you primarily use,’ which was replaced by each participant’s response (e.g., Zoom or Gather.town).

**Intention to Use Virtual Meeting Technology**

1. I intend to use the platform I primarily use for virtual meetings for work in the future.
2. I will likely use the platform I primarily use for virtual meetings for work in the future.
3. I expect that I will use the platform I primarily use for virtual meetings for work in the future.

**Perceived Usefulness and Ease of Use**

**Perceived Usefulness (Im et al., 2011)**

1. I find the platform I primarily use for virtual meetings useful in my life.
2. Using the platform I primarily use for virtual meetings enables me to accomplish tasks more quickly.
3. Using the platform I primarily use for virtual meetings increases my productivity.

**Perceived Ease of Use (Im et al., 2011)**

1. It is easy to become skillful at using the platform I primarily use for virtual meetings.
2. I find the platform I primarily use for virtual meetings easy to use.
3. Learning to operate the platform I primarily use for virtual meetings was easy for me.

**Facial Appearance Dissatisfaction**

**Body Image – Negative Physical Self Scale (Chen et al., 2006):**

**To what extent do you agree with the following statements:**

1. I am depressed about how my face looks.
2. If it is possible, I will change the way my face looks.
3. If there is some way I can improve my face, I will keep trying to do it.
4. I am ashamed about my facial appearance.

**Impression Management in Virtual Meetings**

**Thinking about your meetings for work on the platform you primarily use for virtual meetings, how often do you think the following statements are true:**

1. I use touch-up to enhance my self-video.
2. I use video filters (image overlays that partially cover my self-video image) to change how I appear to others.
3. I use avatars (that fully cover my video image of my face/body) to change how I appear to others.
4. I use filters for my voice so others cannot hear my own voice.

**Virtual Meeting Fatigue**

**Zoom Exhaustion & Fatigue Scale (ZEF Scale) (Fauville et al., 2021):**

**Please answer these questions about your experiences with meetings on the platform you primarily use for virtual meetings:**

1. How tired do you normally feel after meetings on the platform you primarily use for virtual meetings?
2. How exhausted do you normally feel after meetings on the platform you primarily use for virtual meetings?
3. How mentally drained do you normally feel after meetings on the platform you primarily use for virtual meetings?
4. How blurred does your vision normally feel after meetings on the platform you primarily use for virtual meetings?
5. How irritated do your eyes normally feel after meetings on the platform you primarily use for virtual meetings?
6. How much do your eyes hurt after meetings on the platform you primarily use for virtual meetings?
7. How much do you normally feel like avoiding social situations after meetings on the platform you primarily use for virtual meetings?
8. How much do you want to be alone after meetings on the platform you primarily use for virtual meetings?
9. How much do you normally need to take some time by yourself after meetings on the platform you primarily use for virtual meetings?
10. How emotionally drained do you normally feel after meetings on the platform you primarily use for virtual meetings?
11. How irritable do you normally feel after meetings on the platform you primarily use for virtual meetings?
12. How moody do you normally feel after meetings on the platform you primarily use for virtual meetings?
13. How much do you dread having to do things after meetings on the platform you primarily use for virtual meetings?
14. How much do you normally feel like doing nothing after meetings on the platform you primarily use for virtual meetings?
15. How much do you normally feel too tired to do other things after meetings on the platform you primarily use for virtual meetings?

**Virtual Meeting Duration**

**[Daily Work-Related Meeting Duration Hours]**
In an average workday, how many hours do you tend to spend in work-related video meetings on the platform you primarily use for virtual meetings?

- Answer options: 0, 1, 2, 3, 4, 5, ... 19+

**References**

[Im I, Hong S, Kang MS. An international comparison of technology adoption: Testing the UTAUT model. Information & Management. 2011;48: 1–8. doi:](http://paperpile.com/b/C3DEKH/ajW9)[10.1016/j.im.2010.09.001](http://dx.doi.org/10.1016/j.im.2010.09.001)

[Chen H, Jackson T, Huang X. The Negative Physical Self Scale: Initial development and validation in samples of Chinese adolescents and young adults. Body Image. 2006;3: 401–412. doi:](http://paperpile.com/b/C3DEKH/7TwYK)[10.1016/j.bodyim.2006.07.005](http://dx.doi.org/10.1016/j.bodyim.2006.07.005)

[Fauville G, Luo M, Queiroz ACM, Bailenson JN, Hancock J. Zoom Exhaustion & Fatigue Scale. Computers in Human Behavior Reports. 2021;4: 100119. doi:](http://paperpile.com/b/C3DEKH/MkxR)[10.1016/j.chbr.2021.100119](http://dx.doi.org/10.1016/j.chbr.2021.100119)
