# Supplementary material for: Examining attitudes about the virtual workplace: Associations between zoom fatigue, impression management, and virtual meeting adoption intent
Source: PLoS One. 2025 Feb 5;20(2):e0312354. doi: 10.1371/journal.pone.0312354 (PMC11798524; doi:10.1371/journal.pone.0312354)
Supplement: Attachment — Submitted filename: Response to Reviewers.docx [file pone.0312354.s003.docx]

Dear Dr. Anis Eliyana and reviewers,

Thank you for your thoughtful comments and feedback on our work. We have carefully addressed each of the key points raised by the reviewers and incorporated your suggestions to ensure the manuscript aligns with the guidelines of PLOS ONE.

First, to enhance transparency in our research methods, we have added detailed explanations of the data collection process, participant demographics, and the composition of our sample. Additionally, we conducted a statistical analysis to assess the presence of common method variance. The results confirmed that common method bias is not a concern for our study. These details have been included in the Methods and Analysis sections. To further ensure transparency and reproducibility, we have shared the questionnaire items as a supplementary file, along with the R code and data containing the variables of interest for this study.

Second, we have expanded on the theoretical and practical implications of our findings in the Discussion section. Moreover, we added a Limitations and Future Directions section to acknowledge the constraints of our study and propose avenues for future research. We believe these revisions clarify the contributions of our work to the literature and provide practical value for organizations.

In the document below, we have left your comments in blue text and we use black text to denote our responses to reviewer comments and green text for direct quotes from the paper. In the revised submission document (a marked-up copy), we have used green text to denote our main changes.

We hope this approach makes the reviewing process as efficient as possible for you.

Thank you again for your guidance and the opportunity to strengthen our manuscript.

---------------------------------------------------------------------------------------------------------------------

Reviewer #1: Thank you for submitting your paper to PLOS ONE. This is a well-written paper that covers a highly relevant and timely topic. However, there are several areas where improvement is needed to enhance the clarity, depth, and rigor of your study. Please consider addressing the following points:
→ Thank you very much for your positive feedback and recognizing the relevance and timeliness of our study. We greatly appreciate your thoughtful comments and suggestions, which have provided us with valuable insights to improve our manuscript. We have carefully addressed each of the points you raised, as outlined in our detailed responses below.

a) Research Problematization and Contributions: The introduction should clearly articulate the research problem and specific contributions your study makes to the literature. Presenting these aspects more clearly will strengthen the framing and relevance of your research.

→ Thank you for your insightful feedback. We have revised the introduction to more clearly articulate the research problem, identify the gaps in the existing literature, and highlight the specific contributions of our study. We believe these revisions strengthen the framing and underscore the relevance of our research. Please see the updated introduction section for these improvements.

The increasing reliance on virtual meetings (VMs) has led to a pervasive experience of VM fatigue, commonly referred to as “Zoom fatigue” [[1]](https://paperpile.com/c/C3DEKH/MkxR). This phenomenon has significant implications for workplace productivity and individual well-being, as VM fatigue impedes effective engagement with interactions in VMs [[2]](https://paperpile.com/c/C3DEKH/azVi). Notably, women and people of color report higher levels of VM fatigue, potentially stemming from facial dissatisfaction during self-view [[2–4]](https://paperpile.com/c/C3DEKH/tN92+qPVl+azVi). While disabling self-view has been suggested as an effective solution [[5–7]](https://paperpile.com/c/C3DEKH/biIr+6uZy+a1lD), it remains impractical for many users who prefer to self-monitor during meetings. Beyond disturbing workplace interactions and productivity, such negative experiences can create psychological barriers to adopting VM technologies [[8]](https://paperpile.com/c/C3DEKH/OKaN), contributing to technology inequity in the workplace. Despite VM fatigue’s critical role in shaping workplace interactions and digital inclusion in emerging virtual work environments, its effects on VM adoption—and the mechanisms linking facial appearance concerns, VM fatigue, and VM adoption—remain underexplored.

This study responds to the pressing need to understand the mechanism of VM fatigue and its consequences for virtual workplace technology inclusion. Specifically, we investigate impression management features–tools that enable users to adjust their self-video to manage their appearance. By exploring the relationships among facial appearance concerns, VM fatigue, and behavioral intentions toward VM technologies, the present research uncovers what motivates the use of these tools to manage appearance-related concerns and how such usage can promote equity, inclusion, and user satisfaction.

This work’s theoretical contributions include extending the concept of impression management to encompass appearance-related concerns in VM contexts, with broader implications for equity and inclusion in remote work environments. The research also advances understanding of the psychological mechanisms underlying VM fatigue and its influence on technology adoption by drawing upon the Uses and Gratifications (U&G) perspective, Computer-Mediated Communication (CMC) theories (i.e., the Social Information Processing theory and the Hyperpersonal Model), and the Technology Acceptance Model (TAM). By connecting facial appearance dissatisfaction, appearance-related impression management behaviors via VM technologies, and VM fatigue, the study highlights actionable pathways for VM platforms to enhance user well-being and technology adoption.

b) Theoretical Foundation and Hypotheses Development: The theoretical foundation requires further development by **incorporating recent and relevant literature.** This will provide a stronger basis for your hypotheses. Suggested literature includes studies on **technology adoption, such as Adoption of Telecommuting in the Banking Industry**: A Technology Acceptance Model Approach (DOI: https://doi.org/10.28945/5023). Integrating these works will enhance the relevance and currency of your theoretical framework.

→ Thank you for your constructive comments. We have incorporated recent and relevant literature on technology adoption, including the suggested study, to further develop the theoretical foundation and provide a stronger basis for our hypotheses. Additionally, we have updated other sections of the literature review by integrating recent studies on impression management, computer-mediated communication (CMC), and Zoom fatigue to enhance the relevance and currency of our framework. Below, we provide the updated text for the technology adoption section. For updates to other literature review sections, please refer to the main manuscript.

 …The explored determining factors of PU and PEOU in virtual learning and communication contexts include usability, facilitating conditions, playfulness, system quality, enjoyment, and individual differences such as self-efficacy [[90–95]](https://paperpile.com/c/C3DEKH/rEdk+FYPQ+0h5c+3ltL+fTRE+Rdnw)....

A construct similar to VM fatigue, for instance, technostress— the overload and tenseness feeling induced by using novel technologies [[97]](https://paperpile.com/c/C3DEKH/xwAD)—was found to be negatively associated with technology acceptance behaviors [[98–102]](https://paperpile.com/c/C3DEKH/wJUs+gokc+0bPv+RA25+VpgG), potentially resulting in innovation resistance within organizations. Recent literature exploring the adoption of remote teaching technologies showed that technostress undermines PU and PEOU, leading to negative intentions to use the technology [[103]](https://paperpile.com/c/C3DEKH/dB5H). Similarly, anxiety about teleconferencing systems has been shown to reduce PU, thereby decreasing the actual use of these systems [[104]](https://paperpile.com/c/C3DEKH/TEPL). On the contrary, some research highlights the impact of positive experiences with VM technologies. One study found that the intention to adopt Zoom meetings was predicted by the perceived excitement of using the platform [[105]](https://paperpile.com/c/C3DEKH/TEq7), suggesting that positive experiences with VM technologies can promote VM adoption. These findings suggest that while negative experiences with VM technologies can create psychological barriers and hinder VM adoption, positive experiences can foster acceptance and continued use of VM technologies.

c) Methodology: The methodology section can be strengthened by providing more detailed information regarding you**r sample population, the sampling technique employed, and sample adequacy.** Additionally, **including the questionnaire form in the appendix** would add transparency to your study design and improve its validity.

→ Thank you for raising this important point. We have provided additional details regarding our sample population, the sampling technique employed, and sample adequacy to strengthen the methodology section. Furthermore, we have included the questionnaire form in the appendix to enhance transparency and improve the validity of our study design. Please see the updated text for these revisions.

***Participants***

We recruited 2,448 U.S.-based workers to participate in the single cross-sectional 15-minute survey through a research panel provided by Qualtrics. Qualtrics restricted the sample to include professional, technical, or scientific workers who worked remotely at least sometimes and who participated regularly in virtual meetings for work. The sample was intentionally stratified by two gender categories (men and women) and four racial categories (African American, Asian, Hispanic or Latino, and White)—chosen as representative of the most prominent groups in the sample—with approximately even participation among the eight subgroups in order to facilitate statistical comparisons. The survey questions were randomly distributed to the overall sample of 2448 participants to reduce the duration of survey and to minimize measurement errors caused by response fatigue.

d) Analysis: The analysis section is sound, and no major changes are necessary.

→ Thank you for acknowledging our efforts.

e) Theoretical Implications: While you have presented the practical implications well, the **theoretical implications are underdeveloped**. Expanding on how your findings contribute to the theoretical understanding of your topic would provide a more balanced discussion of the study's impact.

→ We appreciate your constructive feedback. To address this, we have expanded the theoretical implications of our study and structured the Discussion section into four distinct parts: theoretical implications for Uses and Gratifications (U&G), Computer-Mediated Communication (CMC) theories, VM fatigue and Technology Adoption Model (TAM), as well as practical implications. For brevity, we have not included the revised Discussion section here but kindly refer to the main manuscript for the updated content.

Thank you very much for your time and constructive feedback.

---------------------------------------------------------------------------------------------------------------------

Reviewer #2: Congratulations to the authors for reaching this stage. The manuscript includes several revision suggestions that should be considered, as follows:
→ Thank you very much for acknowledging our work and providing constructive feedback. Your thoughtful comments have been invaluable in strengthening and refining our manuscript. We have carefully addressed each of your suggestions, as detailed in the responses below.

1. Improving Manuscript Coherence: To enhance coherence, it is necessary to elaborate on previous references and research in the Introduction and Hypothesis Development sections (citation numbers 1-99). In the Discussion section, however, new references are largely introduced (citation numbers 111-117) without prior mention. Therefore, it is suggested that the authors incorporate these new references (citation numbers 111-117) in the Introduction and Hypothesis Development sections.

→ Thank you for bringing this to our attention. We have incorporated most of the references you highlighted into the Hypothesis Development section to improve manuscript coherence. However, we have chosen to retain one study (Edwards et al., 2010, previously citation number 115) exclusively in the Discussion section, as it specifically emphasizes the implications of our findings for virtual meeting technology design. This placement aligns with the study’s relevance to the broader implications of our research.

[Previous citation 111] Additionally, evidence suggests that having greater self-appearance concerns—arising from excessive self-monitoring through self-video streaming in VMs—lead individuals to engage more with self-enhancement features during these meetings [(Pikoos et al. 2021)](https://paperpile.com/c/C3DEKH/pZEr).

[Previous citation 112] Impression management, referred to as the control of information about oneself to shape others’ perceptions ([Schlenker](https://www.annualreviews.org/search?value1=B+R+Schlenker&option1=author&noRedirect=true&sortField=prism_publicationDate&sortDescending=true) & [Weigold](https://www.annualreviews.org/search?value1=M+F+Weigold&option1=author&noRedirect=true&sortField=prism_publicationDate&sortDescending=true), 1992), influences not only how individuals are perceived by others but also satisfaction with meeting interactions (Crowe et al., 2019).

[Previous citation 113, 114]  As physical attractiveness can be a substantial factor influencing how others perceive an individual’s favorable qualities in organizational settings, individuals may become concerned about the impressions they make based on their physical appearance [(Gardner and Martinko 1988; Leary, 2019)](https://paperpile.com/c/C3DEKH/MbTs+N79e).

[Previous citation 115, we kept it only in Discussion section] Technologies interact with a variety of layers within organizations and society at large (Edwards et al., 2010), necessitating the design of VM platforms that effectively address substantial user needs, desires, and behaviors related to VMs.

[Previous citation 116] Indeed, impression management is a fundamental behavioral tactic commonly observed in meeting interactions [(Pernelet and Brennan 2023)](https://paperpile.com/c/C3DEKH/Kq86).

[Previous citation 117] Considering the substantial role of nonverbal cues in signaling participants’ states and attitudes in the VMs [(Peper et al. 2021; Hellmann et al. 2020)](https://paperpile.com/c/C3DEKH/A3s5+5SgR)

[Citations in previous order]

111. [Pikoos TD, Buzwell S, Sharp G, Rossell SL. The zoom effect: Exploring the impact of video calling on appearance dissatisfaction and interest in aesthetic treatment during the COVID-19 pandemic. Aesthet Surg J. 2021;41: NP2066–NP2075. doi:](http://paperpile.com/b/C3DEKH/pZEr)[10.1093/asj/sjab257](http://dx.doi.org/10.1093/asj/sjab257)

112.	[Crowe J, Yoerger M, Harms M, Lehmann-Willenbrock N, Allen JA. Meeting mirth: The critical role of impression management and humor style in meetings. HUMOR. 2019;32: 23–48. doi:](http://paperpile.com/b/C3DEKH/CkXZ)[10.1515/humor-2017-0103](http://dx.doi.org/10.1515/humor-2017-0103)

113.	[Gardner WL, Martinko MJ. Impression Management in Organizations. J Manage. 1988;14: 321–338. doi:](http://paperpile.com/b/C3DEKH/MbTs)[10.1177/014920638801400210](http://dx.doi.org/10.1177/014920638801400210)

114.	[Leary MR. Self-Presentation. Routledge; 2019. Available:](http://paperpile.com/b/C3DEKH/sXw6) <https://play.google.com/store/books/details?id=u9WuyQEACAAJ>

115.	[Edwards WK, Newman MW, Poole ES. The infrastructure problem in HCI. Proceedings of the SIGCHI Conference on Human Factors in Computing Systems. New York, NY, USA: Association for Computing Machinery; 2010. pp. 423–432. doi:](http://paperpile.com/b/C3DEKH/EGmk)[10.1145/1753326.1753390](http://dx.doi.org/10.1145/1753326.1753390)

116.	[Pernelet HR, Brennan NM. Impression management at board meetings: accountability in public and in private. Accounting, Auditing & Accountability Journal. 2023;36: 340–369. doi:](http://paperpile.com/b/C3DEKH/Kq86)[10.1108/AAAJ-09-2022-6050](http://dx.doi.org/10.1108/AAAJ-09-2022-6050)

117.	[Hellmann A, Ang L, Sood S. Towards a conceptual framework for analysing impression management during face-to-face communication. Journal of Behavioral and Experimental Finance. 2020;25: 100265. doi:](http://paperpile.com/b/C3DEKH/5SgR)[10.1016/j.jbef.2020.100265](http://dx.doi.org/10.1016/j.jbef.2020.100265)

2. Separate Paragraph for Discussion on SIP Theory and U&G Theory: The Discussion section should include a distinct paragraph analyzing how the findings confirm and extend both SIP (Social Information Processing) theory and U&G (Uses and Gratifications) theory.

→ Thank you for highlighting this point. We have added two distinct sections to the Discussions to clearly demonstrate our theoretical implications:

- Theoretical implications from the U&G perspective: Understanding workers’ socio-psychological needs in VM interactions
- Theoretical implications: Extending CMC theories, SIP theory and the hyperpersonal model to virtual meetings

We hope these additions clarify how our findings confirm and apply CMC theories (SIP and the hyperpersonal) and the U&G perspective. Please refer to the updated Discussion section in the main manuscript for details.

3. Adding Implications and Limitations: After the Discussion section, an Implications and Limitations section should be added. The Implications should clarify the theoretical and practical contributions of the findings, while the Limitations should indicate that this study is restricted to the U.S. context. Future research could explore other contexts, perspectives, theoretical bases, and factors not addressed in this study.

→ Thank you for raising this point. We have addressed your suggestion by adding an *Implications and Limitations* section following the Discussion. For *Implications*, we included three distinct sections for theoretical contributions and one for practical contributions within the Discussion section. In the *Limitations and Future Directions* section, we explicitly acknowledge that our study is confined to the U.S. context and propose future research directions to explore other contexts not addressed in this study. Please refer to the updated manuscript for these revisions.

4. Enhancing Robustness with Method Variance Testing: To improve robustness, it is recommended to conduct tests addressing common method variance and common method bias issues.

→ We appreciate you raising this important point. To address your feedback, we conducted Harman’s Single-Factor Test to examine common method variance. Additionally, we included explanations of the steps taken in our study procedures to minimize common method bias. Please refer to the following for the updated Methodology and Results sections for details.

*Under Measures section*

To prevent common method bias, we ensured participant anonymity before introducing the survey questions. Additionally, we included unrelated questions that were not tied to our variables of interest to minimize the potential for consistent response patterns caused by priming or consistency effects.

*Under Data Analysis section*

 Prior to the analyses, we conducted Harman’s Single-Factor Test to investigate the existence of significant common method bias. The results indicated that 39% of variance was explained by the single factor model, below the commonly accepted threshold of 50% [(Kock 2020)](https://paperpile.com/c/a5B4wL/wens). This suggests that our data were not significantly affected by common method bias.

5. Consistency in Findings Presentation: For consistency in the presentation of findings, the final two sentences in the Abstract should align with the key points in the Conclusion section. Additionally, the Conclusion should elaborate on the SIP theory.

→ Thank you for your invaluable feedback. We have addressed this comment by revising both the Abstract and Conclusion sections to ensure consistency in the presentation of findings. In the Conclusion, we elaborated on the SIP theory and clarified that our study primarily focuses on one of its core assumptions, as well as the Hyperpersonal Model. Please see the following for details.

**Abstract**

Impression management is a crucial tactic within the workplace milieu. This study establishes a connection between impression management and the negative self-evaluation stemming from heightened self-monitoring during virtual meetings (VM), which manifests in the form of Zoom (VM) fatigue. We conducted a cross-sectional survey, by recruiting 2,448 U.S.-based workers.  Our survey results revealed that facial appearance dissatisfaction is associated with VM fatigue, resulting in lower intention to adopt VM technologies due to decreased perceived usefulness of VM technologies. Furthermore, building upon the Uses and Gratification (U&G) perspective and the assumptions of the Social Information Processing (SIP) theory and the Hyperpersonal Model, our findings illuminate that VM fatigue prompts the use of impression management behaviors by using VM features, closely linked to dissatisfaction with one’s facial appearance. The results suggest that utilization of impression management features in VMs is driven by needs related to facial appearance concerns, which is associated with impression management. This study extends the concept of impression management to VM environments in the workplace, underscoring the importance of addressing workers’ needs and well-being to foster a worker-friendly VM communication environment and promote VM acceptance. This study identifies external factors within the Technology Acceptance Model by integrating the U&G perspective, the SIP theory, and the Hyperpersonal Model to understand the mechanisms underlying VM fatigue and adoption in the emerging virtual workplace.

**Conclusions**

Our study provides insights into the intricate psychological and social repercussions entwined with the adoption and use of VM technology within professional settings. Our study highlights the significance of understanding employees' encounters with technology within multifaceted environments where societal and psychological factors converge with both the users and the technology itself. Particularly, our study contributes to understanding the mechanism of VM fatigue, highlighting that VM fatigue is closely related to individual needs and desires for impression management in workplace VMs. We build on CMC theories, particularly the assumptions of the SIP theory and the Hyperpersonal Model, which suggest that people adaptively harness available cues within communication modalities to facilitate selective self-presentation. Our study demonstrates that users leverage features provided by VM technologies to fulfill their self-presentation needs. By incorporating the TAM and UG perspectives, we highlight that virtual meeting fatigue was related to facial appearance dissatisfaction and reduced perceived usefulness, which impedes technology adoption intent. We suggest that future research should delve deeper into how current VM features facilitate workplace communication in manners that supports the well-being and socio-psychological needs of workers.

These adjustments aim to strengthen the coherence, theoretical contribution, and rigor of the manuscript.

→ We appreciate your time and effort in helping us improve our manuscript.

---------------------------------------------------------------------------------------------------------------------

Reviewer #3: I would like to congratulate the authors on the successful completion of their research and the writing of this article. However, there are several issues I would like to address and suggest for the improvement of this article:

→ Thank you for acknowledging our work and for your constructive feedback. We greatly appreciate your insights and have carefully addressed each of your comments to enhance the quality of our manuscript. Please find our detailed responses below.

1. The authors need to provide a more detailed explanation of the data collection process. Four models were tested with each sample, and there are two points that need clarification:
a) Was the data collection for the four models conducted simultaneously or at different times? If at different times, please explicitly explain the time gap between sample collections for each model.

b) Did the respondents who filled out the questionnaires for models 2, 3, and 4 also fill out the questionnaire for model 1?

→ Thank you for raising these points. We conducted a cross-sectional study in which the survey questions were randomized to minimize the risk of consistent response patterns, as this study included numerous items as part of a larger project. We emphasized and clarified that participants were invited for a single cross-sectional study and that all responses were collected simultaneously. Additionally, we specified whether respondents who answered the questions for Models 2 and 3 were included in Model 1. Please see the following text (by referring to the participants section for further details).

We recruited 2,448 U.S.-based workers to participate in the single cross-sectional 15-minute survey through a research panel provided by Qualtrics. Qualtrics restricted the sample to include professional, technical, or scientific workers who worked remotely at least sometimes and who participated regularly in virtual meetings for work. The sample was intentionally stratified by two gender categories (men and women) and four racial categories (African American, Asian, Hispanic or Latino, and White)—chosen as representative of the most prominent groups in the sample—with approximately even participation among the eight subgroups in order to facilitate statistical comparisons. The survey questions were randomly distributed to the overall sample of 2448 participants to reduce the duration of survey and to minimize measurement errors caused by response fatigue…. Of the 267 participants for Model 2, 39 were excluded from the analyses for Models 1 and 3 because they indicated that impression management features were not applicable to the virtual meeting platform they primarily used. All participants included in Model 3 were included in the Model 1 analysis. As this survey was a single cross-sectional study, all responses for the study's models were collected at the same time.

2. The authors need to add explanations on the procedural steps taken to minimize common method variance.

→ Thank you for highlighting this point. We have added detailed explanations regarding the procedural steps taken to minimize common method variance in our study. Please see the following (Added to the Measures section)

To prevent common method bias, we ensured participant anonymity before introducing the survey questions. Additionally, we included unrelated questions that were not tied to our variables of interest to minimize the potential for consistent response patterns caused by priming or consistency effects.

3. The authors should include an explanation of the statistical measures used to ensure that common method variance is not an issue in this research analysis. This is important considering that data collection for each model was (allegedly) done cross-sectionally and used the same measurement scale for each variable.

→ Thank you for your valuable feedback. To ensure that common method variance was not an issue for our data, we conducted Harman’s Single-Factor Test. The results indicated that no single factor accounted for the majority of variance, confirming that common method variance is unlikely to affect our findings. Please refer to this section for details (Added to the Data Analysis section).

 Prior to the analyses, we conducted Harman’s Single-Factor Test to investigate the existence of significant common method bias. The results indicated that 39% of variance was explained by the single factor model, below the commonly accepted threshold of 50% [(Kock 2020)](https://paperpile.com/c/a5B4wL/wens). This suggests that our data were not significantly affected by common method bias.

4. The authors briefly mention the theoretical implications in the conclusion section. However, they should create a separate section dedicated to theoretical implications, especially focusing on the contribution of this research to the TAM, SIP, and U&G theories.

→ Thank you for raising this important point. We have addressed your suggestion by creating distinct sections dedicated to the theoretical implications under the Discussion section, with a specific focus on contributions to TAM, CMC theories (SIP and the Hyperpersonal Model), and U&G theories. The updated sections are as follows:

- Theoretical implications from the U&G perspective: Understanding workers’ socio-psychological needs in VM interactions
- Theoretical implications: Extending CMC theories, SIP theory and the hyperpersonal model to virtual meetings
- Theoretical Implications: VM Fatigue as a Barrier to Technology Adoption via PU

We hope these additions provide a clearer elaboration of the theoretical implications of our study. Please refer to the updated Discussion section for details.

5. In addition to the theoretical implications, the authors also need to elaborate on the managerial implications aimed at organizations.

→ Thank you for your valuable feedback. We have addressed this comment by adding a distinct subsection titled *Practical Implications* within the Discussion section, where we elaborate on the managerial implications aimed at organizations and recommendations for technology design.

6. The limitations of this research need to be stated explicitly, especially regarding aspects of the research methods. By openly acknowledging the existing limitations, the authors can provide constructive recommendations for future research.

→ Thank you for raising this point. We have added a *Limitations and Future Directions* section to explicitly acknowledge the limitations of our research. In this section, we also provide recommendations for future research to address these limitations.

7. I hope the authors can provide more meaningful and fruitful recommendations for future research. This would enhance the value of the article by offering clear directions for subsequent studies in this field.

→ Thank you for raising this point. We agree that providing meaningful and constructive recommendations for future research enhances the value of our study. In response, we have proposed specific future research directions under each subsection within the Discussion section, linking them to our theoretical and practical implications. As mentioned before,  we have suggested future directions to address the study's limitations within our Limitations and Future Directions section. Please refer to the updated Discussion section for details.
